# Supplementary material for: Assessment of relationship between retinal perfusion and retina thickness in healthy children and adolescents
Source: PLoS One. 2022 Aug 11;17(8):e0273001. doi: 10.1371/journal.pone.0273001 (PMC9371266; doi:10.1371/journal.pone.0273001)
Supplement: S1 Table — (DOCX) [file pone.0273001.s001.docx]

**Table 3.**  Values of the Quasi likelihood Information Criterion (QIC) calculated from GEE analysis of models predicting retinal thickness

| **Model** | **IRT** | | | **MRT** | | | **ORT** | | |
| --- | --- | --- | --- | --- | --- | --- | --- | --- | --- |
|  | Fovea | Parafovea | Perifovea | Fovea | Parafovea | Perifovea | Fovea | Parafovea | Perifovea |
| **SVD** | 10741.01 | 14294.43 | 5932.06 | 39160.47 | 8630.36 | 4265.18 | 26466.99 | 18259.32 | 12264.52 |
| **DVD** | 11673.59 | 19411.52 | 8632.02 | 38197.46 | 8182.67 | 4259.91 | 29168.90 | 18598.74 | 12179.20 |
| **CVD** | 10198.31 | 14782.46 | 7609.72 | 35893.81 | 8629.00 | 4291.99 | 24054.56 | 17680.53 | 12313.70 |
| **SVD, DVD** | 10706.27 | 14292.79 | 5595.67 | 37757.82 | 7771.80 | 4016.16 | 26452.74 | 18065.54 | 12180.32 |
| **SVD, CVD** | 9984.20 | 12847.39 | 5934.26 | 35878.62 | 8619.25 | 4266.17 | 23907.36 | 17669.01 | 12095.94 |
| **CVD, DVD** | 10195.54 | 14782.75 | 7567.39 | 35751.76 | 7813.01 | 4071.46 | 23350.10 | 16847.79 | 11845.20 |
| **SVD, DVD, CVD** | 9927.06 | 12550.91 | 5493.23 | 35756.76 | 7632.69 | 3954.62 | 22866.33 | 16813.07 | 11779.60 |
| **Sex, SVD, DVD, CVD** | 9924.20 | 12454.01 | 4866.91 | 35743.97 | 7622.10 | 3952.81 | 22561.58 | 16714.97 | 11636.71 |
| **Sex, Age, SVD, DVD, CVD** | 9718.58 | 12335.97 | 4868.93 | 34518.21 | 7477.69 | 3953.64 | 22080.05 | 16446.81 | 11612.49 |
| **Sex, Age, BMI, SVD, DVD, CVD** | 9718.99 | 12283.26 | 4855.83 | 34427.16 | 7476.61 | 3934.16 | 22081.56 | 16430.87 | 11602.45 |

Abbreviations: BMI, body mass index; CVD, choriocapillaris vascular density; DVD, deep capillary plexus vascular density; IRT, inner retinal thickness; MRT, middle retinal thickness; ORT, outer retinal thickness; SVD, superficial capillary plexus vascular density
