# Supplementary material for: Assessment of relationship between retinal perfusion and retina thickness in healthy children and adolescents
Source: PLoS One. 2022 Aug 11;17(8):e0273001. doi: 10.1371/journal.pone.0273001 (PMC9371266; doi:10.1371/journal.pone.0273001)
Supplement: Attachment — Submitted filename: PLOS ONE.docx [file pone.0273001.s002.docx]

PONE-D-22-00990R1
Assessment of Relationship between Retinal Perfusion and Retina Thickness in Healthy Children and Adolescents
PLOS ONE

Dear Edrian Nim Tolentino

Thank you for your email.

Actually, I have not received your previous email on Apr 25 2022.

The requested changes were performed as below.

1. Thank you for updating your data availability statement. You note that your data are available within the Supporting Information files, but no such files have been included with your submission. At this time we ask that you please upload your minimal data set as a Supporting Information file, or to a public repository such as Figshare or Dryad.
Please also ensure that when you upload your file you include separate captions for your supplementary files at the end of your manuscript.
As soon as you confirm the location of the data underlying your findings, we will be able to proceed with the review of your submission.

**The data from OCTA VD and thickness was attached as supplementary files.

2. Your ethics statement should only appear in the Methods section of your manuscript. If your ethics statement is written in any section besides the Methods, please delete it from any other section.

**Thanks for the comment. The statement was clarified in the METHOD part and highlighted.

Kind regards,

Fariba Ghassemi MD
