# Supplementary material for: Assessment of relationship between retinal perfusion and retina thickness in healthy children and adolescents
Source: PLoS One. 2022 Aug 11;17(8):e0273001. doi: 10.1371/journal.pone.0273001 (PMC9371266; doi:10.1371/journal.pone.0273001)
Supplement: Attachment — Submitted filename: response to the editors (1).docx [file pone.0273001.s003.docx]

Dear Dr. Alfred Lewin,

Thank you for your review. We revised the last version of article entitled as “**Assessment of Relationship between Retinal Perfusion and Retina Thickness in Healthy Children and Adolescents** - [PONE-D-22-00990R1], addressing the points raised during the review process.

The sample size and statistical analysis were reviewed by our epidemiologist (Dr Siamak Sabour) and the needed corrections were done accordingly. If there is anything else we can do, please let us know.

Sincerely yours,

Fariba Ghassemi MD

PONE-D-22-00990R1

Reviewer #1: I was invited to review the revised version of the manuscript entitled "Assessment of Relationship between Retinal Perfusion and Retina Thickness in Healthy Children and Adolescents". Authors partially responded to my previous observations:
- Sample size calculation seems to be not appropriate. Authors stated "The sample size was calculated as 97 eyes with a 95% confidence interval, 10% of margin of error, and 50% population proportion". From which population? why 50% of proportion? Authors should deeply explain the calculation;

** Dear Reviewer

Thanks for your valuable comments. We learned a lot from these comments. For your information, we consulted with our epidemiologist (Dr Siamak Sabour) and will respond according to his opinion. In the continuity of our work on normal variations of vascular density of adult eyes, we were asked to do the same study in the eyes of children. The first purpose of the study was to evaluate the vascular density of the eyes that was a quantitative type of data which were reported ***as percentage***.

The above mentioned 10% of the change in the vascular density would be considered as significant. It was better to be said 10% of the change in the **unit** (per percent). The “Power and Sample Size Calculation Software version 3.1.2” was used for evaluating the appropriateness of sample size as another software. You can see the results below.

We are planning a study of a **continuous** response variable from independent control and experimental subjects with 1 control(s) per experimental subject. In a previous study the response within each subject group was normally distributed with standard deviation of 10. If the true difference in the experimental and control means is 10 **units**, we will need to study 17 eyes in each age categories to be able to reject the null hypothesis that the population means of the experimental and control groups are equal with probability (power) 0.80. The type I error probability associated with this test of this null hypothesis is 0.05.

We think that from our sentence in the rebuttal letter as “10% of margin of error, and 50% population proportion", you have considered it as a dichotomous outcome analysis with the results shown below. But our analysis was a descriptive and analytic test on **quantitative parameters only**. In the case of using such dichotomous outcome analysis, we needed to have a sample size by 376.

Although, we lost some cases due to the limitation of CORONA crisis in the course of the study, according to the used sample size within each age group (by nearly 24 cases in each 4 age groups of studied children and adolescents ranged from 3 to 18 YO), the power was calculated to be 92%.

We were planning a study of subjects in which we will regress their values of yvar against xvar. Prior data indicate that the standard deviation of xvar is 0.1 and the standard deviation of the regression errors will be 0.2. If the true slope of the line obtained by regressing yvar against xvar is 0.5, we will need to study 10 subjects to be able to reject the null hypothesis that this slope equals zero with probability (power) 0.0.80. The Type I error probability associated with this test of this null hypothesis is 0.05.

- It is unclear were Authors performed ANOVA and KWs tests;

**Thanks a lot for meticulous attention. We used ANOVA and KW tests for evaluating our four age groups of children, because our parameters were quantitative parameters in our four aging groups as that reported from our previous study on these children and adolescents.

“Ghassemi F, Hatami V, Salari F, Bazvand F, Shamouli H, Mohebbi M, Sabour S. Quantification of macular perfusion in healthy children using optical coherence tomography angiography. Int J Retina Vitreous. 2021 Oct 2;7(1):56. doi: 10.1186/s40942-021-00328-2. PMID: 34600586; PMCID: PMC8487563.”

This study is a second look for the data, evaluating the correlation between the thickness and the vascular density in macular area.

- The lack of sensitivity parameters should be highlighted in limitation section.

**Thanks for the comment. This is added as “In addition, sensitivity parameter analysis was not performed in this analysis” to the limitation section.

- P-values correction for multiple comparisons was not performed in correlation analysis reported in table 2.

** The correction was done according the Bonferroni method and the needed change was performed in the result part and highlighted.

Minor observations:
- Foveal SVD in table 1 was reported as mean and SD;

**As stated in the legend of the table, the variables with normal distribution are presented with mean ± standard deviation and non-normally distributed data presented with median (interquartile range or min - max).

- The interquartile range should be reported as (Q1; Q3);

**Thanks, it is corrected.

- Table 3 should be shifted to supplementary material;

**Thanks, Table 3 shifted to supplementary material.
